# La tuberculose abdominale pseudo-tumorale

**Published:** 2012-10-15

**Authors:** Rachid El Barni, Mohamed Lahkim, Abdessamad Achour

**Affiliations:** 1 Service de chirurgie générale. Hôpital militaire Avicenne, Marrakech, Maroc

**Keywords:** Tuberculose, pseudo-tumorale, abdomen, Tuberculosis, pseudotumorale, abdomen

## Abstract

**Introduction:**

L’objectif de ce travail est de rapporter cinq cas de tuberculose abdominale pseudo-tumorale afin d’en souligner les aspects diagnostiques et thérapeutiques. Cinq observations sont colligées dans le service de chirurgie générale de l’hôpital militaire Avicenne de Marrakech au cours de l’année 2007. Les aspects cliniques sont disparates. Ainsi, les auteurs ont noté un syndrome péritonéal dans un cas, une masse épigastrique dans un cas, une lésion suspect du sigmoïde dans un cas, une masse de la fosse iliaque droite dans un cas et une altération de l’état général avec fièvre dans le dernier cas. Un seul patient avaient bénéficié d’une biopsie scano-guidée et les quatre patients restants avaient été opérés. Une masse du méso côlon était notée dans le premier cas. Dans le second cas, l’aspect de la masse épigastrique et son siège avaient orienté vers une tumeur du grand omentum. Une localisation tuberculeuse péritonéale et sigmoïdienne avait été trouvée dans le troisième cas. Le diagnostic d’une tumeur du côlon droit était hautement suspect chez le patient séropositif qui avait présenté une péritonite post-opératoire et décédé à J + 3 dans un tableau de choc septique. Le siège et l’aspect nécrotique des lésions trouvées à la tomodensitométrie chez la seule patiente de l’étude avaient fait discuter en premier un lymphome. Même en l’absence d’antécédents de tuberculose pulmonaire, le diagnostic tuberculose abdominale pseudo-tumorale doit être évoqué surtout dans un pays d’endémie comme le notre et le recours à une laparotomie est justifié chaque fois que persiste un doute diagnostique ou en cas de complication.

## Introduction

La tuberculose extra-pulmonaire représente près de 1/3 des cas de tuberculose déclarés au Maroc [1]. Elle siège par ordre de fréquence décroissant, au niveau ganglionnaire, génito-urinaire, ostéo-articulaire et neuro-méningé [2]. La localisation abdominale est relativement fréquente et représente 5 à 10% de l’ensemble des localisations [3].

La symptomatologie est non spécifique et la palpation d’une masse abdominale peut orienter à tort vers une pathologie tumorale maligne, d’autant plus que la symptomatologie évolue dans un contexte d’altération de l’état général. Ce diagnostic, difficile et fréquemment méconnu, doit être évoqué surtout si le contexte épidémiologique s’y prête [3], en présence d’une atteinte pulmonaire concomitante, ou devant des antécédents de tuberculose.

Les formes pseudo-tumorales de la tuberculose abdominale sont rarement rapportées dans la littérature, engendrant un retard diagnostique et par conséquent la persistance de l’évolutivité de cette maladie normalement curable. Les auteurs ont donc jugé utile de rapporter cinq cas de tuberculose abdominale pseudo-tumorale et d’en souligner les aspects diagnostiques et thérapeutiques.

## Patients et observations

### Observation 1

NW. S, âgé de 18 ans, sans antécédents pathologiques particuliers, avait présenté depuis trois jours des douleurs abdominales diffuses et maximales en péri-ombilical sans syndrome occlusif. A son admission, le patient était fébrile à 39°, son état hémodynamique était stable. La palpation abdominale trouvait une sensibilité abdominale diffuse avec une défense para-ombilicale droite. Le toucher rectal était indolore et le doigtier revenait propre.

Le taux des globules blancs était à 19 500 éléments/mm3. Une radiographie de l’abdomen sans préparation objectivait une aérocolie. L’échographie abdominale trouvait un épanchement péri-cæco-appendiculaire et au niveau du douglas sans épaississement appendiculaire.

**Figure 1:**
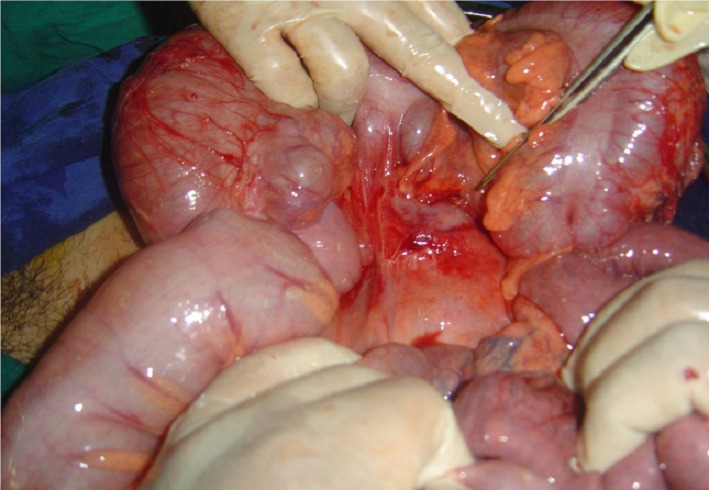
Vue opératoire montrant une masse mal limitée du méso côlon en regard de l’angle colique droit

Une laparotomie exploratrice avait été décidée et qui avait trouvé un appendice normal, un épanchement péritonéal séreux de moyenne abondance avec une masse dure mal limitée au niveau du méso côlon en regard de l’angle colique droit (Figure 1). À son ouverture, cette masse avait un contenu purulent et une coque épaisse. Un prélèvement du pus, des biopsies multiples de la coque, une toilette et un drainage de la cavité abcédée ont été réalisés. Les suites opératoires ont été simples et l’examen anatomo-pathologique des biopsies de la coque revenait en faveur d’une tuberculose péritonéale. Une chimiothérapie anti-tuberculeuse a été instaurée et l’évolution était favorable au bout de deux mois.

### Observation 2

**Figure 2:**
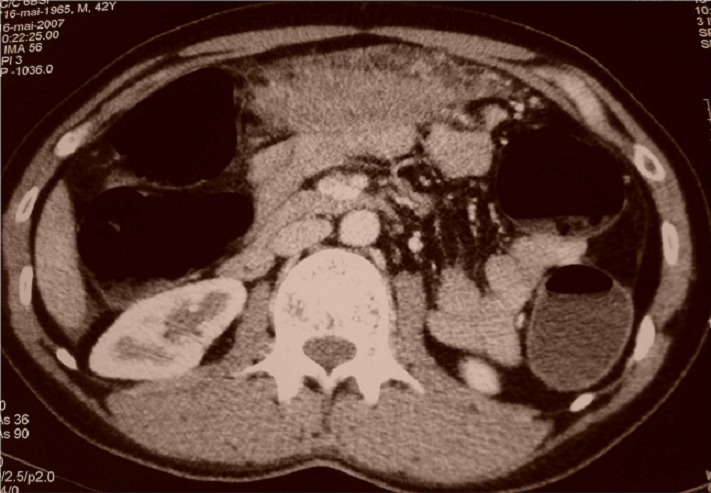
Coupe tomodensitométrique montrant une masse du grand omentum associée à une distension colique avec stase stercorale

**Figure 3:**
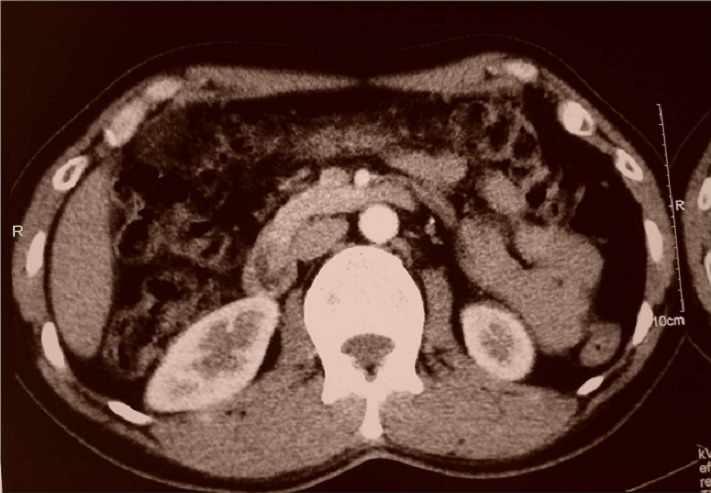
Coupe tomodensitométrique montrant un nettoyage complet de la lésion du grand omentum après 3 mois de traitement anti-tuberculeux

M.O, âgé de 42 ans, ayant comme antécédent pathologique un tabagisme chronique, souffrait depuis trois semaines de douleurs épigastriques associées à un syndrome subocclusif avec fièvre et altération de l’état général. L’examen clinique trouvait un patient fébrile à 38,5° avec présence d’une masse épigastrique ferme et sensible. La biologie montrait un syndrome inflammatoire non spécifique et une hyperleucocytose à 12 700 éléments blancs/mm3. La tomodensitométrie (TDM) abdominale objectivait une masse ovalaire d’environ 10 cm de grand axe, comportant quelques images arrondies de tailles variables spontanément hypodenses et se rehaussant de façon hétérogène. Cette masse attenante au grand omentum, était associée à une distension colique avec stase stercorale (Figure 2). Elle avait orienté, du fait de son aspect et de son siège vers une tumeur du grand omentum de type tumeur stromale, mésothélium malin ou tuberculose dans sa forme pseudo-tumorale. L’intradermo-réaction (IDR) à la tuberculine était faiblement positive. Une ponction scano-guidée a était réalisée et l’examen anatomo-pathologique avait conclu a un aspect morphologique vaguement granulomateux avec nécrose caséiforme soulevant le diagnostic de tuberculose. Une chimiothérapie anti-tuberculeuse a été instaurée et l’évolution clinique était bonne avec nettoyage complet de la lésion du grand omentum au bout de 3 mois de traitement anti-tuberculeux (Figure 3).

### Observation 3

**Figure 4:**
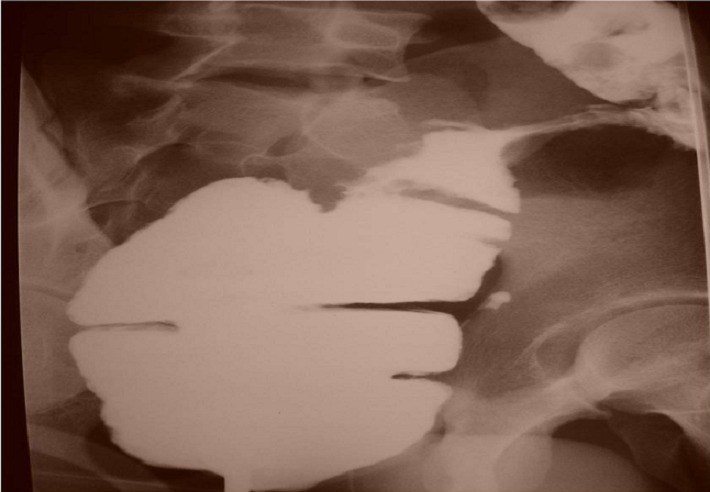
Lavement à la gastrografine montrant un aspect en «trognon de pomme» du côlon sigmoïde

B.M, âgé de 23 ans, sans notion de contage tuberculeux, présentait depuis deux mois des douleurs abdominales diffuses avec trouble du transit à type de diarrhée glaireuse sans rectorragie évoluant dans un contexte d’asthénie, d’anorexie et d’amaigrissement chiffré à 10 kg. L’examen clinique à l’admission trouvait un patient apyrétique en mauvais état général, avec un abdomen distendu et une sensibilité de la fosse iliaque gauche sans organomégalie et le toucher rectal était normal. Les aires ganglionnaires périphériques étaient libres. La radiographie du thorax avait montré une pacchypleurite axillaire droite avec scissurite. La radiographie de l’abdomen sans préparation trouvait une aérocolie sans niveau hydro-aérique. Un lavement à la gastrografine a mis en évidence une lésion végétante étendue sur 4 cm et siégeant au niveau du côlon sigmoïde se raccordant en trognon de pomme avec le reste du côlon (Figure 4). La colonoscopie avait confirmé la présence d’une lésion ulcéro-bourgeonnante au niveau du côlon sigmoïde réduisant la lumière colique et évoquant une tumeur colique dont la biopsie était en faveur d’une colite granulomateuse sans nécrose caséeuse ni signes de malignité. L’échographie abdominale avait montré un épanchement liquidien en interanses, et la TDM abdomino-pelvienne avait objectivé en plus une stase stercorale colique en rapport avec épaississement pariétal circonférentiel et irrégulier du sigmoïde. Les examens biologiques ont montré une anémie microcytaire à 9,6 g/dl et un syndrome inflammatoire non spécifique. L’IDR à la tuberculine et la recherche des mycobactéries dans les crachats étaient négatives. La sérologie HIV était négative et les marqueurs tumoraux (antigène carcino-embryonnaire, antigène carbo-hydrate 19-9) avaient des taux normaux.

Une laparotomie exploratrice était décidée. L’exploration chirurgicale avait trouvé un épanchement péritonéal séreux, des nodules péritonéaux et une distension colique en rapport avec une sténose du sigmoïde. Les gestes réalisés étaient une biopsie des nodules péritonéaux, une sigmoïdectomie avec anastomose colorectale. L’examen anatomo-pathologique des biopsies péritonéales et de la pièce de sigmoïdectomie était en faveur d’un processus inflammatoire granulomateux spécifique largement nécrosé évoquant une tuberculose. Les suites opératoires étaient simples. Une chimiothérapie anti-tuberculeuse a été préconisée pendant une année au bout de laquelle le patient avait repris du poids avec disparition de tous les signes cliniques.

### Observation 4

**Figure 5:**
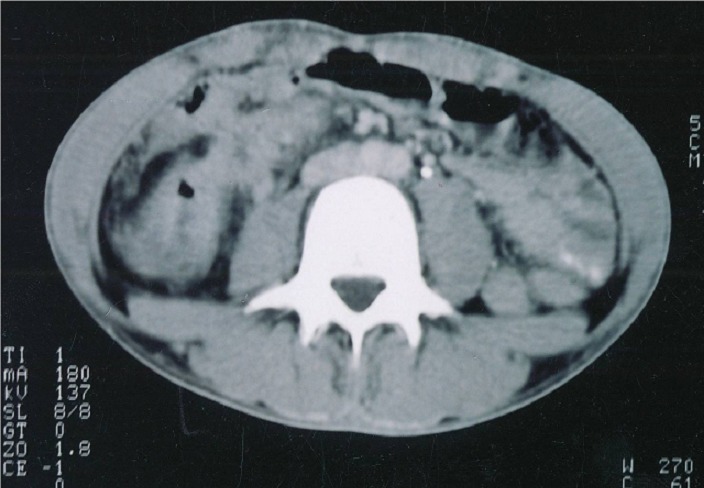
Coupe tomodensitométrique montrant une masse du côlon droit à contours irréguliers

**Figure 6:**
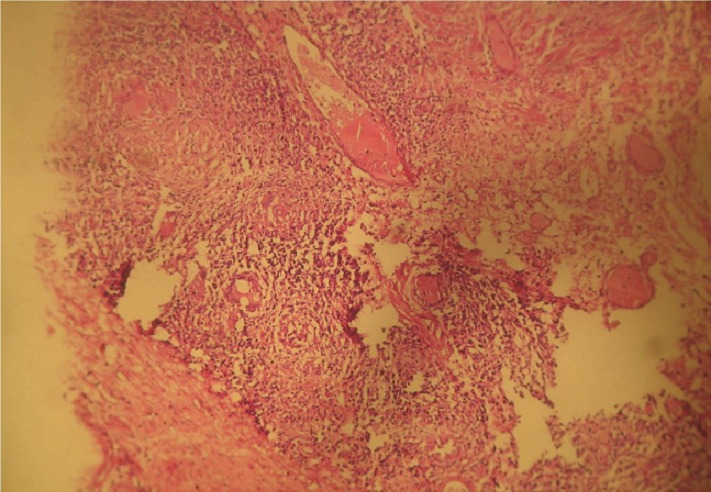
Granulome giganto-cellulaire jouxtant la musculeuse colique (HE X 40)

M.M, âgé de 26 ans, connu séropositif, était hospitalisé pour masse douloureuse de la fosse iliaque droite (FID) et des troubles du transit intestinal à type d’alternance diarrhée-constipation évoluant depuis trois mois. L’examen abdominal avait trouvé une masse de la FID ferme, douloureuse, adhérente au plan profond et mobile par rapport au plan superficiel mesurant six cm de diamètre. Le reste de l’examen clinique ne trouvait pas d’hépato-splénomégalie ni d’adénopathies périphériques et le toucher rectal était sans particularité. Le bilan biologique a révélé un syndrome inflammatoire non spécifique. L’échographie complétée par la TDM abdominale avaient montré une masse de la FID mesurant 5×6 cm à structure solide, à contours irréguliers, à développement surtout intra-colique, et qui se rehaussait après injection du produit de contraste (PC) (Figure 5). En outre, on notait l’absence d’ascite, d’adénopathies profondes et de lésions hépatiques. Devant cet aspect, l’origine néoplasique a été retenue et l’origine inflammatoire a été évoquée en dernier lieu. Les marqueurs tumoraux (ACE, AFP, CA 19-9) étaient normaux. Au cours de son hospitalisation, le patient avait présenté un syndrome subocclusif et une laparotomie était décidée en urgence et qui avait permis la réalisation d’une résection iléocæcale avec anastomose iléo-transverse termino-latérale. Les suites opératoires étaient marquées par une péritonite à J + 2 ayant nécessité une reprise chirurgicale et la réalisation d’une double stomie. Le patient était décédé à J + 3 dans un tableau de choc septique. L’examen anatomo-pathologique qui a conclu à une tuberculose colique pseudo-tumorale (Figure 6).

### Observation 5

**Figure 7:**
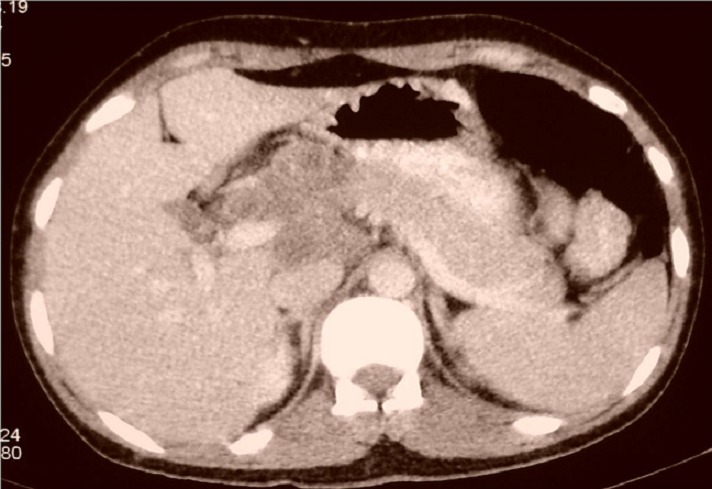
Coupe tomodensitométrique montrant une image vraisemblablement nécrotique de la région coelio-mésentérique englobant l’isthme du pancréas

N. Z âgée de 38 ans, infirmière, hospitalisée pour altération de l’état général évoluant depuis un an avec fièvre. L’examen clinique avait trouvé une patiente fébrile à 38,5° et qui présentait une masse épigastrique ferme et sensible. La biologie avait montré un syndrome inflammatoire non spécifique et l’IDR à la tuberculine était à 12 mm. La TDM abdominale avait montré une image spontanément hypodense, se rehaussant en périphérie après injection du PC, vraisemblablement nécrotique, centrée sur la région coelio-mésentérique, englobant l’isthme du pancréas et s’étendant au hile hépatique et à l’arrière cavité des épiploons (Figure 7). Cette image était associée à d’autres images de même nature rétro-péritonéales, péri-aorto-caves et iliaques notamment à droite. Cet aspect faisait discuter une hémopathie en premier lieu et en particulier un lymphome. Le diagnostic de tuberculose pseudo-tumorale avait été également évoqué. La biopsie d’une adénopathie iliaque droite était réalisée et l’examen anatomo-pathologique avait conclu à une tuberculose ganglionnaire. L’évolution clinique était favorable au bout de 6 mois de chimiothérapie anti-tuberculeuse.

## Discussion

L’incidence de l’infection tuberculeuse a connu une ré-ascension non seulement dans les pays en voie de développement mais aussi dans les pays développés. Ceci est expliqué en partie par l’infection au virus de l’immunodéficience acquise (VIH), la précarité et l’immigration [2]. Ce qui a entrainé une augmentation de l’incidence des localisations extra-pulmonaires qui représentent près de 1/3 des cas de tuberculose déclarés au Maroc [1]. La localisation abdominale constitue une forme extra-pulmonaire relativement fréquente, elle représente 5 à 10% de l’ensemble des localisations [3]. Cette fréquence est plus importante et pourrait doubler à tripler chez les sujets séropositifs. Elle a été estimée à 13,5% dans une étude faite sur 199 patients séropositifs [3, 4]. Au cours de la tuberculose abdominale tous les organes peuvent être atteints, et les localisations les plus habituelles de la tuberculose digestive sont l’intestin grêle (44%), le caecum (35%) et l’iléo-caecum (16%) [5]. L’atteinte isolée du côlon est rare, elle est estimée entre 2 et 9% et dominée par l’atteinte du côlon droit [6]. Les autres localisations abdominales concernent les ganglions, le péritoine, le foie et la rate [7]. Il s’agit d’une localisation péritonéale dans la première observation, d’une localisation au niveau du grand omentum dans la deuxième observation, d’une localisation péritonéale et sigmoïdienne qui est une association plus rare faisant la particularité de cette observation, d’une localisation colique droite chez le patient séropositif et d’une localisation ganglionnaire diffuse chez la seule patiente de l’étude.

L’atteinte digestive peut être primitive par ingestion directe de mycobactérium ou secondaire à des lésions pulmonaires très bacillifères par voie hématogène ou lymphatique [8]. L’agent bactérien est le plus souvent le bacille de Kokh bovin ou humain, exceptionnellement ce sont les mycobactéries atypiques chez les sujets immunodéprimés [8]. La forme hypertrophique pseudo-tumorale serait le plus souvent primitive [7]. Cette forme touche l’adulte jeune entre 20 et 40 ans [10]. La prédominance féminine est retrouvée dans les localisations intestinales [11]. Dans nos observations, il s’agit de quatre hommes âgés successivement de 18 ans, 42 ans, 23 ans et 26 ans. Notre patiente était âgée de 38 ans.

La fréquence des formes pseudo-tumorales au cours de la tuberculose abdominale est difficile à évaluer [2]. En effet, un aspect pseudo-tumoral n’est signalé que dans 5% des cas [3].

La symptomatologie des tuberculoses du tube digestif diffère, bien entendu, de celle des tuberculoses péritonéales qui se manifestent par des douleurs abdominales et surtout une ascite [12, 13]. C’est le cas de la première observation. Le tableau clinique de la tuberculose digestive est en général peu spécifique, se traduisant par un amaigrissement (80%), une fébricule (66%), un ballonnement abdominal douloureux (100%), une constipation (40%), une ascite (40 à 100%), une diarrhée (15%) avec parfois un syndrome dysentérique en cas de localisation recto-sigmoïdienne, voire une forme pseudo-tumorale (5%) [14]. Des complications sont à craindre dont la perforation, le plus souvent cloisonnée, l’occlusion, l’hémorragie ou la malabsorption en cas d’atteinte iléocæcale [14].

Les perturbations biologiques observées n’ont d’autre part rien de spécifique, qu’il s’agisse de l’anémie ou du syndrome inflammatoire. L’intradermo-réaction (IDR) à la tuberculine n’est pas une preuve de la nature tuberculeuse de l’affection. La radiographie du thorax peut être utile pour attester de l’atteinte secondaire [1].

Les examens complémentaires sont d’une utilité limitée, sauf s’il existe une forte suspicion du diagnostic [15]. Les radiographies d’abdomen sans préparation dans les syndromes subocclusifs ou occlusifs ne font que confirmer l’existence d’un obstacle. Sur les opacifications du tube digestif, les sténoses (Figure 4) ont la même morphologie que celle que l’on peut observer dans la maladie de Crohn [3], diagnostic, à priori, beaucoup plus probable dans les pays industrialisés [16]. Grâce à l’échographie et à la TDM, l’imagerie constitue une étape décisive quant au diagnostic des formes pseudo-tumorales [7]. L’atteinte intestinale, prédomine au carrefour iléocæcal et se présente comme des anses agglomérées, à paroi épaissie de façon habituellement concentrique [7]. L’épaississement pariétal habituellement concentrique peut, quand il est excentré et à développement exophytique extrinsèque, simuler plus une atteinte tumorale qu’une atteinte inflammatoire [7]. Cet épaississement peut être hétérogène avec des foyers hypodenses en rapport avec de la nécrose caséeuse [7]. Le diagnostic de tuberculose reste difficile, et l’atteinte du carrefour iléocæcal peut simuler d’autres affections telles la maladie de Crohn, une néoplasie, ou une tumeur appendiculaire [7]. L’atteinte tuberculeuse peut aussi intéresser n’importe quel autre segment du tube digestif. Elle est souvent représentée par des anses agglutinées, une infiltration pariétale digestive hypertrophique avec des nodules péritonéaux, et un amas d’adénopathies profondes notamment mésentériques. Mais cet aspect peut manquer, et devant une infiltration digestive hypertrophique irrégulière excentrée, une origine tumorale est souvent évoquée [3]. La localisation ganglionnaire dans la tuberculose intra-abdominale peut également être à l’origine de l’aspect pseudo-tumoral. Il peut s’agir d’un amas d’adénopathies de petite taille agglomérées satellites d’une atteinte digestive, péri-pancréatiques ou pédiculaires hépatiques comme le cas de la patiente de notre étude. Ce groupement pourtant spécifique de la tuberculose oriente plus souvent, du fait du siège de la lésion vers une pathologie tumorale [17].

La tuberculose des organes pleins, dans sa forme hépato-splénique macro-nodulaire unique ou multiple, peut simuler pour une lésion tumorale maligne primitive ou secondaire [17]. Des formes exceptionnelles de tuberculose péritonéale telles la forme fibro-adhésive et la forme ulcéro-caséeuse peuvent aussi donner un aspect pseudo-tumoral [3]. La forme fibro-adhésive, pose quant à elle le diagnostic différentiel avec les tumeurs carcinoïdes [7]. L’apport de l’IRM dans cette localisation abdominale est non spécifique, cet examen montre des lésions en hyposignal T1 avec un signal variable T2 au cours des localisations ganglionnaires et viscérales [7]. Devant un aspect hautement suggestif de l’imagerie, un bilan étiologique devrait être entamé pour dépister d’autres localisations pouvant conforter le diagnostic. Si le doute persiste une ponction guidée sous contrôle échographique ou scanographique avec étude histologique permettrait de poser le diagnostic [3].

En définitive, si l’urgence et la symptomatologie clinique permettent de les réaliser sans risque, les examens endoscopiques sont les plus utiles [18]. En effet, l’apport de l’endoscopie est essentiel, elle permet la détection des lésions, même les plus superficielles [9, 19]. Son intérêt majeur est qu’elle permet la réalisation de biopsies avec études histologique et bactériologique (culture) et évite alors la morbidité et la mortalité liées à une laparotomie exploratrice [20, 21]. La coloscopie peut montrer soit des érosions muqueuses, des ulcérations avec des zones nécrotico-hémorragiques, soit des zones de rétrécissement ou de sténose infranchissable avec des bourgeons autour [22]. Cependant, les biopsies per-endoscopiques ne sont pas toujours concluantes. Des colorations spécifiques (Ziehl-Nilson) et une mise en culture seront systématiquement réalisées (positive dans 42 à 69%) [20].

Plus récemment décrite, la recherche du BK par « polymerase chain reaction » (PCR) sur biopsies permet une forte sensibilité diagnostique (75 à 80%) et une spécificité élevée (85 à 95%) [20].

Savoir évoquer le diagnostic de tuberculose est la condition indispensable pour une prise en charge rapide et adaptée, car le pronostic vital est mis en jeu [3]. La laparotomie reste parfois le seul recours en cas de négativité de la ponction écho ou scano-guidée [3]. En effet, 20 à 40% des malades [6] subiront une laparotomie, soit en urgence devant une complication (sténose, occlusion, masse compressive, mise à plat de certaines cavités caséifiées, perforation et fistule), soit dans un but diagnostique. Le traitement percutané peut être proposé en cas de formes abcédées [23].

Le traitement chirurgical n’est pas standardisé et dépend d’abord du motif de l’indication opératoire. Ainsi, la levée d’un ou de plusieurs obstacles en cas d’occlusion ou un procédé d’hémostase en cas d’hémorragie massive vont rendre habituellement nécessaire une résection. Il dépend aussi et surtout des lésions constatées à l’exploration chirurgicale et qui impliquent le plus souvent des résections intestinales avec ou sans rétablissement de la continuité ou des dérivations internes ou des stomies. Le choix d’une résection doit toujours prendre en compte l’étendue de la résection. Si elle devait être trop étendue, surtout au niveau de l’iléon, des stomies doivent être préférées en comptant sur le traitement antituberculeux pour réduire l’étendue de résections secondaires qui seraient nécessaires [24]. Les constatations per-opératoires ne permettent pas toujours de reconnaître la nature tuberculeuse de ces formes pseudo-tumorales et le diagnostic ne sera définitivement posé qu’après examen histologique de la pièce opératoire [1].

Ce traitement chirurgical doit être associé à un traitement antituberculeux et c’est le cas de tous nos patients. La nature des complications postopératoires ne diffère pas de celle que l’on peut observer dans d’autres indications. En revanche, leur fréquence élevée comme la mortalité postopératoire s’expliquent par le fait qu’il s’agisse de malades tuberculeux, venant souvent consulter tardivement, dans un état général très altéré [24].

## Conclusion

La tuberculose abdominale pseudo-tumorale, rare même en pays d’endémie comme le notre, pose un grand problème diagnostique notamment avec le cancer. Ce qui justifie souvent le recours à une laparotomie chaque fois que persiste un doute diagnostique ou encore en cas de complication.
